# The Complex Biodiversity-Ecosystem Function Relationships for the Qinghai-Tibetan Grassland Community

**DOI:** 10.3389/fpls.2021.772503

**Published:** 2022-01-27

**Authors:** Wei Qi, Xiaomei Kang, Johannes M. H. Knops, Jiachang Jiang, A. Abuman, Guozhen Du

**Affiliations:** ^1^State Key Laboratory of Grassland Agro-ecosystems, School of Life Sciences, Lanzhou University, Lanzhou, China; ^2^Department of Health and Environmental Sciences, Xi’an Jiaotong Liverpool University, Suzhou, China; ^3^Gansu Provincial Extension Station of Grassland Techniques, Lanzhou, China

**Keywords:** biodiversity, community assembly, ecosystem function, functional diversity, phylogenetic diversity, specific leaf area, seed mass, Tibetan grassland

## Abstract

Despite the long history of the study of the biodiversity-ecosystem function relationship, uncertainty remains about the relationship of natural grassland ecosystems under stressful conditions. Recently, trait- and phylogenetic-based tests provide a powerful way to detect the relationship in different spaces but have seldom been applied to stressful zones on a large spatial scale. We selected Qinghai-Tibetan as the study area and collected a grassland community database involving 581 communities. We calculated biomass and species’, functional, and phylogenetic diversity of each community and examined their relationships by using linear and non-linear regression models. Results showed an overall positive biodiversity-productivity relationship in species’, functional and phylogenetic space. The relationship, however, was non-linear, in which biodiversity explained better the variation in community biomass when species diversity was more than a threshold, showing a weak effect of biodiversity on ecosystem function in low species diversity communities. We also found a filled triangle for the limit of the relationship between species and functional diversity, implying that functional diversity differs significantly among communities when their species diversity is low but finally converges to be a constant with increasing communities’ species diversity. Our study suggests that multiple niche processes may structure the grassland communities, and their forces tend to balance in high-biodiversity communities.

## Introduction

The worldwide biodiversity decline has led to an increasing scientific interest in examining how biodiversity changes affect ecosystem functioning (Hooper et al., 2005; Cadotte et al., 2009). A well-supported hypothesis is that plant species differ in traits that generate different abilities to exploit resources, such as light, water, nutrients, pollinators, etc. Thus, because of different traits, with higher species richness a plant community is able to use more resources, increase productivity, and thereby ecosystem functioning (Liu et al., 2015; Duffy et al., 2017).

However, species richness may be a poor proxy for biodiversity, because the number of plant species often cannot capture the diversity in species traits that determines total resource use (Chalcraft et al., 2009). Therefore, the current research focus has shifted to other aspects of diversity. Functional diversity may quantify trait differences that define species interactions better (Reiss et al., 2009; Rosenfield and Müller, 2020), and therefore capture how the diversity within a community influences both resource acquisition (Violle et al., 2007) and community productivity (Chollet et al., 2014). However, questions remain about which traits need to be considered, and how to translate trait differences into ecological differences (Cadotte et al., 2009; Cadotte, 2017). Another alternative approach is to use the phylogenetic distances among co-occurred species to calculate a phylogenetic relatedness index. Functional trait dissimilarity is correlated with species evolutionary divergence time (Cadotte et al., 2009; Cadotte, 2017), thus a community phylogenetic relatedness index may be a good way to capture the functional, evolutionary, and ecological dissimilarity among species (Kraft et al., 2007; Cadotte et al., 2009; Lemoine et al., 2015). However, the explanatory power of phylogenetic diversity is largely dependent on the species pool and is often questioned because inter-specific functional or niche differences are rarely caused by their evolutionary divergence (Chollet et al., 2014; Lososová et al., 2016). Therefore, recent research focus has shifted to integrating aspects of species richness, functional and phylogenetic diversity and such integrated methods are more effective in capturing variation along environmental gradients in ecosystem productivity, and community structure (Cadotte et al., 2009; Bu et al., 2014; Liu et al., 2015; Rolo et al., 2016; Lyu et al., 2017; de la Riva et al., 2018).

The relationship between species richness and either functional or phylogenetic diversity can also provide insight into the mechanism driving ecosystem functioning and community assembly (Micheli and Halpern, 2005). A positive linear relationship indicates that each species has unique traits or is phylogenetic distinct. Such a positive relationship implies that functional or phylogenetic diversity is complementary among species and that each species contributes equally to ecosystem functioning. In contrast, the absence of a relationship implies functional redundancy, reflecting that species share similar traits or are closely related (Micheli and Halpern, 2005; Mensah et al., 2018). Non-linear relationships imply that the relationship between species richness and either functional or phylogenetic diversity varies depending on species richness (Guerrero et al., 2014). For example, a logarithmic relationship between species richness and functional diversity implies that functional diversity is strongly positively correlated with species richness at low species richness levels, and much less at higher species richness levels (Micheli and Halpern, 2005; Bu et al., 2014). Furthermore, the pattern of this relationship may depend on which functional traits are selected. For instance, Lososová et al. (2016) found a positive correlation of species richness and functional diversity in dispersal traits (dispersal type, seed mass, and seed bank type) and competitiveness traits (plant height, SLA, and LDMC). However, in contrast, there was no relationship for species richness in relation to niche trait preferences for light, temperature, soil moisture, and nutrients. Therefore, different kinds of traits, reflecting different aspects of plants’ function, have to be combined to gain a better understanding of the overall relationship (de la Riva et al., 2018).

The species, functional and phylogenetic aspects of diversity have been examined in many plant communities where environmental stress is non-significant (Hooper et al., 2005; Chalcraft et al., 2009; Jaillard et al., 2014; Zhu et al., 2016; Duffy et al., 2017). In these communities, competition for resources dominates species interactions and functional redundancy among species is common because a small number of dominant species with similar optimal traits structure the communities (thus functional clustering; Micheli and Halpern, 2005; Kang et al., 2015; Mensah et al., 2018; Rosenfield and Müller, 2020; but seen in Mayfield and Levine, 2010, they have assumed in theory that competition for resources may drive functional/phylogenetic over-dispersion or clustering because species have traits contributing to both competitive ability and niche differences). However, in some stressful ecosystems, facilitation, i.e., positive interactions between plant species, have been documented (Callaway et al., 2002; Michalet et al., 2014; Fichtner et al., 2017). Functional dissimilarity is essential for positive species interactions (Maestre et al., 2009; Lyu et al., 2017), thus functional complementation among species should be expected. However, even in communities where facilitation occurs, competition is also frequently documented. Thus, examining the relationships between species richness and functional or phylogenetic diversity in a stressful ecosystem, e.g., Qinghai-Tibetan Plateau (QTP), where both competitive and facilitative plant interactions, as well as meantime significant environmental filtering, operate (Choler et al., 2001; Anthelme et al., 2014; He and Bertness, 2014), may provide new insights into species coexistence and ecosystem functioning.

Alpine/subalpine grasslands are common on the eastern and central QTP covering ∼1.282 million km^2^, or ∼13.4% of China’s total area. These high-altitude grasslands are exposed to extreme weather conditions and vary dramatically in plant richness, abundance, productivity, species composition, and plant traits along environmental gradients (Wu and Chen, 2004; Yang et al., 2009; Qi et al., 2021). This large variability may give rise to the significant differences in species’ interactions among communities with different structure traits or under different environmental conditions (Choler et al., 2001; Callaway et al., 2002; Schöb et al., 2013; Michalet et al., 2014; Lyu et al., 2017), resulting in a possibly significant shift in the relationships among species richness, productivity, and plant functional traits. Recently, in QTP grasslands the relationship between productivity and different aspect of diversity have been observed in a few small-scale observational studies (Zhu et al., 2015; Zhang et al., 2018) and a dozen manipulative studies, such as fertilization (Niu et al., 2014; Li et al., 2015), mowing or grazing management (Niu et al., 2016), and planting or removing individuals (Liu et al., 2015; Lyu et al., 2017). The often conflicting findings about how the diversity affects community productivity (e.g., positive species diversity effect on productivity in Lyu et al., 2017; Zhang et al., 2018, but negative effect in Niu et al., 2014) as well as which aspects of biodiversity and which kinds of traits or trait combination (e.g., plant height in Li et al., 2015; Liu et al., 2015, but specific leaf area and leaf dry matter content in Zhu et al., 2016) are more important in explaining community productivity, however, call for a large-scale study involving different aspects of relationships to reveal universal patterns of the natural ecosystem.

Here, we collected a large dataset encompassing 40% of the QTP area to examine relationships between different aspects of diversity and community productivity as well as between species richness and plant functional or phylogenetic diversity. We hypothesize that (1) species are largely complementary in stressful QTP environments, thus we predict a positive linear relationship between species richness and productivity; (2) species’ functional dissimilarity is hypothesized to be essential for facilitation, and therefore, we hypothesize that functional diversity will be more effective than species richness in explaining grassland productivity of QTP where facilitative interactions are significant (Choler et al., 2001; Anthelme et al., 2014); (3) the relationship between species richness and either functional or phylogenetic diversity may be complicated because facilitative interactions call for functional or phylogenetic complementary among species, resulting in a positive relationship, or strong environmental filtering enhancing functional or phylogenetic similarity among species, resulting in a non-linear relationship. In addition, we examined the relationship between species richness or community productivity and functional diversity of different dimensions (i.e., stem, leaf, seed, and their combinations) to assess whether the relationship is independent of selected traits.

## Materials and Methods

### Study Area

This study was conducted along seven transects (four west-east and three north-south transects) is mainly alpine and subalpine grasslands located across the eastern and central part of the Qinghai-Tibetan Plateau. Due to the restrictions of terrain and available roads, the transects’ shape appears to be irregular and non-linear. The study area is 1,220 km long and 1,070 km wide, covering a geographic area within the latitude of 29.14–38.84° N and longitude of 88.15–101.51° E.

### Field Sampling and Trait Measurement

**FIGURE 1:**
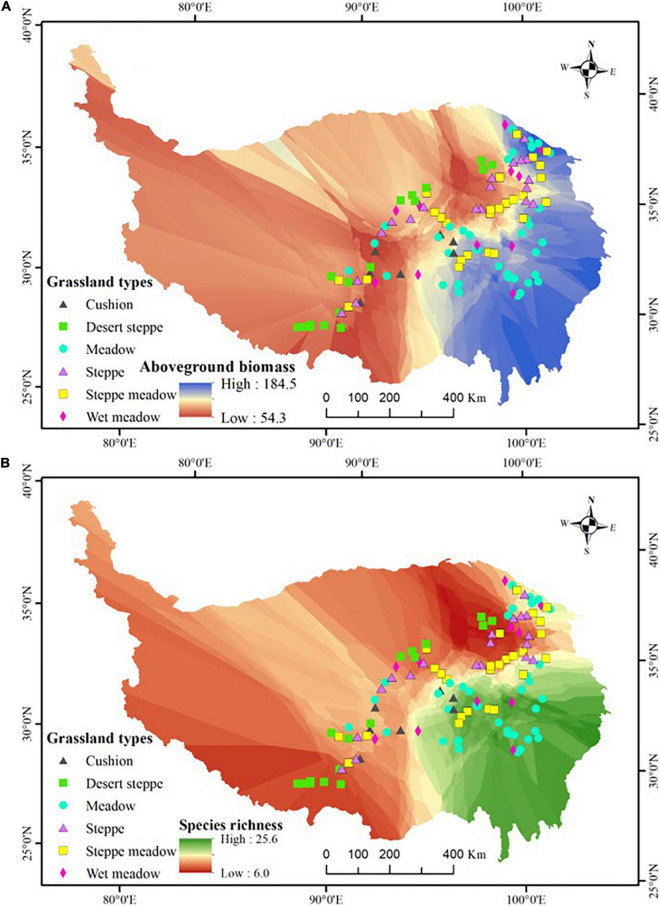
The geographic variation in aboveground biomass **(A)** and species richness **(B)**, and the distribution of the 118 study sites. The map showed the outline of the Qinghai-Tibetan Plateau, which was edited and generated with ArcGIS 10.2 software, http://www.esri.com/. Coordinate system: E, east longitude; N, north latitude.

Field surveys were conducted during late July to early September in 2014 and 2017 and 118 sites were finally surveyed along the seven transects according to the criterion of every 50–60 km interval unless there were no grassland vegetations (e.g., forest, shrub, salt marsh, barren and stony land, lake, farmland, or residential area) (Figure 1 and Supplementary Appendix 1). Field time is the peak aboveground biomass and cover time for most species in the vegetation (Zhu et al., 2015, 2016). All sampling sites were used as yak and sheep winter grazing pasture (from late autumn to early spring) and no obvious summer grazing by livestock or rodents was observed. In total, 118 sites were selected which were as follows: 17 desert steppes, 19 steppes, 26 steppes meadow, 37 meadows, 12 wet meadows, and 7 cushion vegetation (altogether six grassland types). Moreover, seven plots were in the subglacial belt, 35 in the subalpine (including cold temperate), and 76 in the alpine belt. These sites represent an elevational range from 2,792 to 5,217 m, with the climatic range covering a gradient in mean annual temperature (MAT) of -9.04 to 6.07°C and mean annual precipitation (MAP) of 44–716 mm. For selected sites, the number proportion of each grassland vegetation or climate belt is roughly equal to its area proportion in the studied zone (Wu, 1980). In total, we recorded 719 angiosperm species which included almost all dominant species and more than 65% common species in the study region (Wu and Chen, 2004) and we used 719 as the regional species pool number.

For each site, we recorded the geographical coordinates, elevation, slope gradient, and aspect. Five 1 m × 1 m quadrats were laid at a distance of 30–40 m from each other within each 100 m × 100 m site and all vascular plant species were recorded. In the meadow, wet meadow and cushion vegetation individual plants have a very high abundance (up to 2,200/m^2^), thus, for aboveground biomass and abundance, we sampled a smaller0.5 m × 0.5 m quadrat located within the 1 m^2^ quadrats and calculated each species 1 m^2^ abundance and biomass. In total, we sampled 581 quadrats due to the loss of plant material or recording problems in 9 quadrats of 6 sites.

For the vegetation measurements, in each quadrat we recorded the canopy cover, species number, individual plant number, height, and the percent cover and aboveground biomass of each species. Each species aboveground biomass was clipped to the soil level and then weighed after removing any dead parts (standing dead and litter) and dried at 75°C for 48 h to a constant weight.

We selected four traits tightly associated with plant life strategy and functional tradeoff: specific leaf area (SLA), leaf size, plant height, and seed mass. SLA is correlated with other plant traits such as leaf nitrogen content, leaf lifespan, relative growth rate, representing, thus, a trade-off between the leaf carbon acquisition rate and the longevity of plant tissues (Schöb et al., 2013; Qi et al., 2014; Lemoine et al., 2015; Rolo et al., 2016). Plant height and leaf size indicate a trade-off between the competitive or interception ability for light and support structure (e.g., stem, twig, and petiole biomass construction) cost; while seed mass signifies the trade-off between dispersal probability and a seedling establishment’s ability (Cadotte et al., 2009; Niu et al., 2014; Qi et al., 2015; Wagg et al., 2017). Thus, community-level distribution patterns (e.g., clustering or divergence) for these traits can disentangle the importance of these trade-offs during community assembly and help predict how assembly processes respond to environmental changes (Lemoine et al., 2015; Qi et al., 2015; Schmitz et al., 2015; Rolo et al., 2016; Wagg et al., 2017). There were 7,905 species records (samplings) in 581 quadrats (average 13.61 species per quadrat). For each sampling, 3–5 mature individuals and 5–20 fully developed but not senescing leaves were randomly chosen to measure plant height and leaf traits, respectively. The seed mass of most species was from a large database based on our previous studies. The methods of trait measurement referred to Qi et al. (2014, 2015), and their details are described in Supplementary Appendix 2.

### Species Diversity, Phylogenetic Diversity, and Functional Diversity

For each quarter, we first calculated each species’ important value index (IVI) as the average of its relative biomass, relative cover, and relative density. We used IVI instead of the frequently used relative density because the former (1) represents more clearly the status and performance of species in a community, and (2) standardizes the data and makes the variance independent of the mean, which is better for this study with obvious differences in species composition and community structure among sites. Species diversity in each quarter was estimated with both species’ richness and the Shannon–Wiener index [H = –Σ (p_*i*_ × ln p_*i*_), where p_*i*_ is the IVI of species i].

Rao’s quadratic index (FD_*Q*_) was used to estimate the community functional diversity. FD_*Q*_ is the sum of the pairwise trait distances between species weighted by their IVI. FD_*Q*_ for single and multiple traits was calculated by using R and FDiversity (Casanoves et al., 2011). We used Rao’s quadratic index (FD_*Q*_) for two reasons. First, Carboni et al. (2013) has advocated that this measure is more comprehensive for describing the community trait diversity. Secondly, FD_*Q*_ can be used to examine both single and multiple trait space, therefore better to predict variations in ecosystem functions (Butterfield and Suding, 2013).

We calculated the plant community phylogenetic diversity, which was assessed on the basis of a published angiosperm supertree (Phylomatic tree R20120829)^1^. Branch lengths were assigned using the BLADJ algorithm in Phylocom 4.2 software (Webb et al., 2008). To quantify community phylogenetic diversity, we calculated the mean pairwise phylogenetic distance (MPD) using the “construct” function in Phylocom 4.2 software (Webb et al., 2008). The calculation of MPD is mathematically similar to FD_*Q*_, and thus, the combination of MPD and FD_*Q*_ can provide a uniform and effective assessment of functional and phylogenetic community patterns. Based on the phylogenetic tree, we also calculated the phylogenetic signal of each plant trait, and the methods and results of the calculation were referred to in Supplementary Appendix 3.

Species number and abundance varied dramatically among sampled quadrats in our data. To remove any effect of sample size, we calculated the standardized effect size (SES) of MPD and FD_*Q*_ against a null model by generating 10,000 random assemblages for each community, while preserving the species IVI and shuffling the taxon names. For MPD, we calculated SES_*M*_ as: SES_*M*_ = (MPD_*OBS*_ – MPD_*RANDOM*_)/sd(MPD_*RANDOM*_), where sd(MPD_*RANDOM*_) is the standard deviation of the random MPD values. For FD_*Q*_, we calculated SES_*FD*_ as: SES_*FD*_ = (FD_*OBS*_ – FD_*RANDOM*_)/sd(FD_*RANDOM*_). A positive/negative SES_*FD*_ or SES_*M*_ indicates that traits or phylogeny are dispersed/clustered within a community. The difference in species, functional, and phylogenetic diversity among six grassland types is shown in Supplementary Appendix 4, in which meadow had highest species richness, aboveground biomass, SES_*M*_ and SES_*FD*_ of leaf size, SLA, and multiple traits, steppe had highest SES_*FD*_ of plant height and seed mass, desert steppe had lowest aboveground biomass, species richness and SES_*FD*_ of SLA, the wet meadow had lowest SES_*M*_ and SES_*FD*_ of seed mass, and cushion vegetation had lowest SES_*FD*_ of leaf size, plant height, and multiple traits.

### Statistical Analyses

Data on functional traits, aboveground biomass, and species richness were log-transformed to improve normality. We used regression analyses to test for the relationship among aboveground biomass, species diversity (species richness and H), and various functional and phylogenetic diversity indexes. For all regressions, we examined the fit of linear, quadratic, and piecewise models using stat and segmented packages from Software R 4.2.1, and identified the best fit model based on the explained variance (higher R^2^) and parameter significance (lower *P*-values). We constrained the maximum number of breakpoints to one per model to avoid overfitting. In piecewise models, the significance of the difference in regression slope between either side of breakpoint was evaluated by using Davies’ test. In addition, we used the difference in AIC (Akaike information criterion) value (ΔAIC) to compare among models. If ΔAIC was < 5 between linear and non-linear (quadratic and piecewise) models, models were considered not different (Segura et al., 2015), and the simple linear model was selected as the best model.

Communities’ phylogenetic or functional diversity may be structured by multiple niche processes (see the “Introduction”), resulting in a potential high variance among communities even with similar species diversity. Thus, there may be more than a single slope (i.e., rate of change) describing the relationship between species and phylogenetic (or functional) diversity (de Carvalho and Tejerina-Garro, 2015). Accordingly, we then used quantile regression analysis with phylogenetic or each functional diversity index as the response variable. The analysis can identify the limits, boundaries, and shifting within our bivariate distributions by estimating slopes not only through the median but also through each quantile (or percentile) of the relationship. We examined the nature of this upper (lower) bound with quantile regressions with data points in the 0.95 (0.05) quantile using Quantreg package (Koenker, 2018) for R. The significance of the slopes of quantile regressions was assessed with bootstrapped standard errors (with 999 permutations; Silva et al., 2016).

## Results

Structure traits varied dramatically among communities, with species richness ranging from 3 to 37 species, aboveground biomass from 12.4 to 327.64 g, and individual density (abundance) from 4 to 2,252 in a 1 m^2^ quarter (Supplementary Appendix 1). Similar trends were found in various diversity indexes, in which H ranged from 0.58 to 3.13, SES_*M*_ from –4.54 to 1.84, and SES_*FD*_ of leaf size, SLA, plant height, seed mass, and multi-traits from –3.43, –3.00, –2.86, –3.22, and –2.39, to 3.21, 2.18, 3.31, 2.76, and 2.32, respectively. Moreover, based on 95% confidence intervals (CI), the mean SES_*M*_ (95% CI: –0.541 to –0.385, same below) was significantly lower, while the mean SES_*FD*_ of leaf size (0.108–0.278), plant height (0.189–0.407) and multi-traits (0.051–0.184) were significantly but slightly higher than zero, indicating an overall phylogenetic clustering and a functional overdispersion within a community. SES_*FD*_ of SLA (-0.032 to 0.115) and seed mass (–0.120 to 0.034), however, were non-significantly different from zero (Supplementary Figure 1).

**FIGURE 2:**
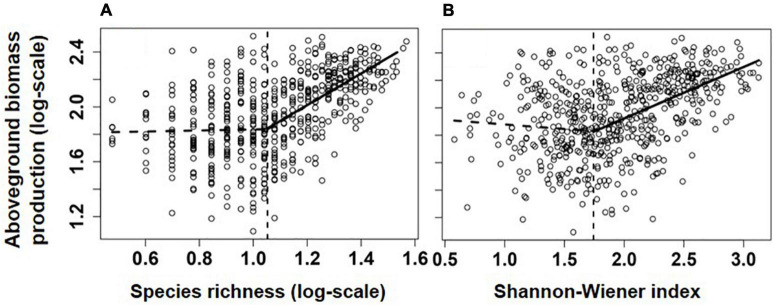
Relationships between aboveground biomass production (log-scale, y-axis, g/m^2^) and species richness **(A)**, species/m^2^) and Shannon-Weaver index **(B)**. Lines showed the best models fitting the relationship, and their significance and explanatory power were shown in Table 1.

**FIGURE 3:**
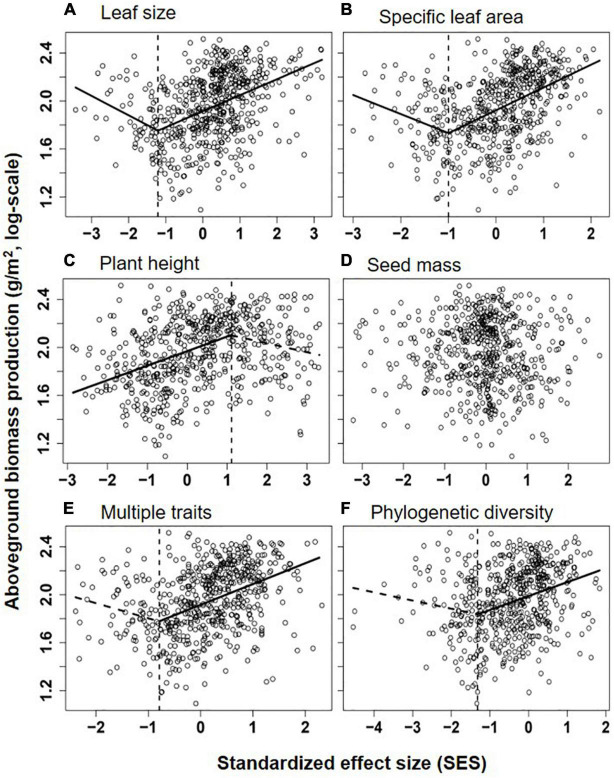
Relationships between aboveground biomass production (log-scale, y-axis) and the standardized effect size (SES) of Rao’s quadratic diversity (FD_*Q*_) for leaf size **(A)**, specific leaf area (SLA, **B)**, plant height **(C)**, seed mass **(D)**, multiple traits **(E)**, and phylogenetic diversity (MPD, **F)**. Lines showed the best models fitting the relationship (the segmented line in piecewise regression was shown as dotted lines when its slope was non-significantly different from zero), and their significance and explanatory power were shown in Table 1.

Overall, communities’ aboveground biomass significantly increased with species diversity (species richness and H), and the best fit model for both relationships was the piecewise regression model, in which the breakpoint was found at about 11.2 species/m^2^ (log-scale species richness = 1.05; 95% CI: 0.94–1.15) and H = 1.74 (1.51–1.97), with the ABP varying non-significantly before the breakpoint and increasing dramatically with species richness or H after the breakpoint (Table 1 and Figure 2). Besides, the fit model for the relationship between ABP and SES_*FD*_ of most of plant traits except for seed mass was also piecewise regression, in which ABP firstly decreased slowly but significantly with SES_*FD*_ of leaf traits and then increased rapidly from the breakpoint at SES_*FD*_ of leaf size and SLA equal to −1.21 and −1.00, respectively, but ABP firstly increased rapidly with SES_*FD*_ of plant height and then varying non-significantly after the breakpoint (Table 1, Figure 3). Finally, the fit model for the relationship between ABP and SES_*M*_ and SES_*FD*_ of multi-traits were similar, they both showed non-significant relationship before the breakpoint and significantly positive relationship after the breakpoint (Table 1, Figure 3).

**TABLE 1:** A comparison of linear, quadratic, and piecewise regression models for the relationship between various (species, functional and phylogenetic) diversity indexes (x-axis) and aboveground biomass production (ABP, y-axis) of the Tibetan grassland communities.

Diversity indexes (x-axis)	Linear				Quadratic				Piecewise			
	Shape	*R* ^2^	Sig.	ΔAIC	Shape	*R* ^2^	Sig.	ΔAIC	Shape (Breakpoint)	*R* ^2^	Sig.	ΔAIC
Species richness	Increase	0.246	<0.001	47.87	U-type	0.296	<0.001	9.97	**Unchange + Increase (1.05)**	**0.310**	**<0.001**	**0**
Shannon–Wiener (H)	Increase	0.166	<0.001	29.83	U-type	0.201	<0.001	7.18	**Unchange + Increase (1.74)**	**0.214**	**<0.001**	**0**
SES_*FD*_ of leaf size	**Increase**	**0.130**	**<0.001**	**27.75**	U-type*	0.140	<0.001	22.99	Decrease + Inchange* (−1.21)	0.177	<0.001	0
SES_*FD*_ of SLA	**Increase**	**0.160**	**<0.001**	**36.52**	U-type*	0.193	<0.001	15.16	Decrease + Inchange* (–1.00)	0.217	<0.001	0
SES_*FD*_ of plant height	Increase	0.089	<0.001	28.66	Unimodal	0.112	<0.001	15.73	**Increase + Unchange (1.11)**	**0.139**	**<0.001**	**0**
SES_*FD*_ of seed mass	Unchange	< 0.001	0.768	0	Unimodal	0.001	0.659	1.25	–	–	–	–
SES_*FD*_ of multi-traits	**Increase**	**0.119**	**<0.001**	**22.05**	U-type*	0.127	<0.001	18.55	Unchange + Increase* (–0.79)	0.157	<0.001	0
SES_*M*_ (SES of MPD)	**Increase**	**0.057**	**<0.001**	**13.63**	U-type*	0.067	<0.001	9.36	Unchange + Increase* (–1.32)	0.085	<0.001	0

*“Shape” column showed the overall shape of the binary regression line, while “R^2^” and “Sig.” columns represented the explanatory power and its significance of regression models, respectively. The shape of the regression line before and after breakpoint (x value; it was a log value of species/m^2^ for species richness, but a dimensionless quantity for other diversity indexes) in piecewise models was arranged on both sides of the plus sign (+). For every binary relationship, a model with the lowest AIC (Akaike information criterion) value was assigned as ΔAIC = 0. “Unchange” meant that the slope of the (segmented) regression line was non-significantly different from zero, while “-” indicated no piecewise regression model suitable for the relationship. In the piecewise regression, it slope for species richness, Shannon–Wiener index, SES_FD_ of leaf size, SLA, plant height, multi-traits and SES_M_ were 0.04 (95% CI: −0.17, 0.25; same below), −0.07 (−0.20, 0.06), −0.16 (−0.28, −0.04), −0.16 (−0.27, −0.04), 0.12 (0.09, 0.15), −0.13 (−0.26, 0.01) and −0.07 (−0.15, 0.02) before the breakpoint, but 1.16 (0.94, 1.38), 0.37 (0.30, 0.45), 0.13 (0.11, 0.16), 0.19 (0.16, 0.22), −0.07 (−0.14, 0.01), 0.17 (0.14, 0.21) and 0.12 (0.08, 0.15) after the breakpoint, respectively. The best fit model was shown in bold.*

There was no consistent relationship between species richness and functional and phylogenetic community diversity. For example, the best model fitting the relationship related to SES_*M*_ and SES_*FD*_ of leaf size, SLA, and multi-traits was also the piecewise model (Table 2) with estimated species diversity thresholds (about 9–11 species/m^2^ for different traits, details were seen in Table 2) slightly lower than those estimated to aboveground biomass. Moreover, the piecewise model was still the optimal model suitable for the relationship between species richness and SES_*FD*_ of plant height, with SES_*FD*_ value firstly decreasing significantly but slowly and then increasing rapidly when species richness was more than the breakpoint (about 12 species/m^2^). On the contrary, the species richness-SES_*FD*_ of seed mass relationship was obscure, and not suitable for any regression model (Table 2).

**TABLE 2:** A comparison of linear, quadratic, and piecewise regression models for the relationship between species richness (x-axis) and six functional and phylogenetic community diversity indexes (y-axis).

Diversity indexes (y-axis)	Linear				Quadratic				Piecewise			
	Shape	*R* ^2^	Sig.	ΔAIC	Shape	*R* ^2^	Sig.	ΔAIC	Shape (Breakpoint)	*R* ^2^	Sig.	ΔAIC
SES_*FD*_ of leaf size	Increase	0.188	<0.001	17.56	U-type	0.214	<0.001	1.12	**Unchange + Increase (0.98)**	**0.218**	**<0.001**	**0**
SES_*FD*_ of SLA	Increase	0.284	<0.001	18.59	U-type	0.306	<0.001	2.70	**Unchange + Increase (0.95)**	**0.311**	**<0.001**	**0**
SES_*FD*_ of plant height	Increase	0.007	0.038	16.70	U-type	0.035	0.001	2.27	**Decrease + Increase (1.08)**	**0.042**	**<0.001**	**0**
SES_*FD*_ of seed mass	Unchange	0.006	0.070	0	U-type	0.008	0.084	0.33	–	–	–	–
SES_*FD*_ of multi-traits	Increase	0.108	<0.001	23.38	U-type	0.143	<0.001	1.90	**Unchange + Increase (0.96)**	**0.149**	**<0.001**	**0**
SES_*M*_ (SES of MPD)	Increase	0.197	<0.001	17.04	U-type	0.215	<0.001	5.80	**Unchange + Increase (0.98)**	**0.225**	**<0.001**	**0**

*A comparison of linear, quadratic, and piecewise regression models for the relationship between species richness (x-axis) and six functional and phylogenetic community diversity indexes (y-axis). “Shape” column showed the overall shape of the binary regression line, “unchange” meant that the slope of the (segmented) regression line was non-significantly different from zero, while symbols (i.e., +, –) and abbreviations (i.e., AIC, ΔAIC, Sig., R^2^) were as specified in Table 1. Species richness breakpoint in piecewise regression models was a log value of species/m^2^ (i.e., 0.95 ≈ 9 species/m^2^). The slope of the piecewise regression for the relationships between species richness and SES_FD_ of leaf size, SLA, plant height, multi-traits, and SES_M_ were −0.08 (95% CI: −1.25, 1.08; same below), 0.40 (−0.69, 1.49), −1.32 (−2.34, −0.29), −0.69 (−1.24, 0.26) and 0.20 (−0.86, 1.25) before the breakpoint, but 3.03 (2.38, 3.69), 2.94 (2.47, 3.41), 2.56 (1.14, 3.98), 2.14 (1.61, 2.67) and 2.88 (2.29, 3.48) after the breakpoint, respectively. The best fit model was shown in bold.*

**FIGURE 4:**
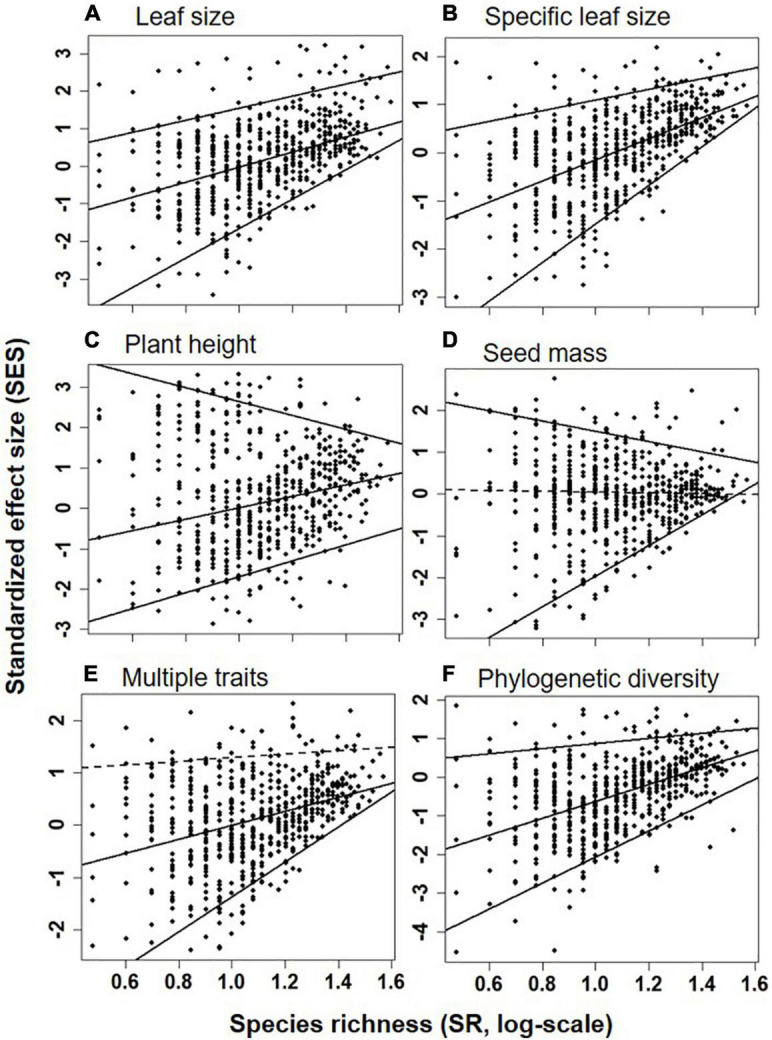
SES of FD_*Q*_ for leaf size **(A)**, specific leaf area **(B)**, plant height **(C)**, seed mass **(D)**, multiple traits **(E)**, and phylogenetic diversity **(F)** in relation to the species richness (SR, log-scale, species/m^2^, x-axis) at the upper (95th), median (50th), and lower (5th) quantile levels. Positive (or negative) SES value indicated greater (or lower) functional and phylogenetic diversity than null. Significant and non-significant linear relationships (at α = 0.05) were shown as solid and dashed lines, respectively.

Quantile regression showed a significant shift in linear regression coefficient (sign and slope) among the upper (95th), median (50th), and lower (5th) quantiles for the relationship between species diversity and functional and phylogenetic diversity (Supplementary Table 1). For example, the relationships of species richness (Figure 4) or H (Supplementary Figure 2) were significantly and strongly positive to SES_*M*_ and SES_*FD*_ of all traits at the lower (5th) quantile, significantly and strongly or weakly positive to SES_*M*_ and SES_*FD*_ of leaf size, SLA, plant height and multi-traits but non-significantly positive to SES_*FD*_ of seed mass at the median (50th) quantile, and significantly but weakly positive to SES_*FD*_ of leaf traits and SES_*M*_, significantly negative to SES_*FD*_ of plant height and seed mass, but non-significantly positive to SES_*M*_ and SES_*FD*_ of multi-traits at the upper (95th) quantile.

## Discussion

Instead of considering species richness as the only facet of diversity, phylogenetic and functional diversity can be widely considered a proxy for ecological differentiation, influencing the structure and composition of communities and thus ecosystem process and function. Consistent with the general pattern, we found an overall positive biodiversity-productivity relationship in species’, functional and phylogenetic metrics (Jaillard et al., 2014; Rolo et al., 2016; Duffy et al., 2017). Our study also demonstrated that species diversity explained better the variation in aboveground biomass only when it was more than a diversity threshold, thereby implying that low species diversity cannot drive the variation of community function. The boundaries of the bivariate distribution of the relationship between species richness or Shannon–Wiener index (H) and SES_*M*_ and single- and multi-trait SES_*FD*_ formed a filled triangle, showing convergence in the limit of the community’s functional and phylogenetic divergence with increasing species diversity. Combined, these results indicate that different assembly processes, such as niche-based deterministic processes like environmental filtering and biotic interactions, and spatial-based neutral processes like dispersal limitation, may structure these communities.

### Overall Patterns in Functional and Phylogenetic Community Diversity

Our analysis evidenced an overall pattern of strongly phylogenetic clustering, but weakly functional overdispersion in the individual and multivariate trait space. The strongly phylogenetic clustering appears to be a result of a stochastic process, such as dispersal limitation. Tibetan Plateau is characteristic of high elevation and a series of huge mountains, which restricts the spread of alpine plants, resulting in the coexistence of closely related plant species with similar evolutionary history. However, to maintain the coexistence and avoid inter-specific resource competition because of similar ecological adaptation, these related species may undergo trait shifts (“character displacement”; Schluter, 2000), thereby reducing niche overlap. Some studies have evidenced that, in many cases, closely related species coexist in close proximity, but are well-separated ecologically and functionally (Lemoine et al., 2015; Qi et al., 2015; Lyu et al., 2017). Moreover, a degree of random functional trait distribution within the phylogeny may also contribute to the inconsistency between functional and phylogenetic community patterns to some extend (Kraft et al., 2007). For instance, the diversity (i.e., mean SES_*FD*_ value) of leaf size and plant height, the traits whose variation are restricted less by phylogeny (based on traits’ phylogenetic signal, seen in Supplementary Appendix 3), have more significant differences to phylogenetic diversity (i.e., mean SES_*M*_ value) in this study.

### The Relationship Between Biodiversity and Grassland Productivity

Our study supports hypothesis 1, i.e., an overall positive relationship between aboveground biomass and species’, functional and phylogenetic diversity, indicating the important role of biodiversity at different aspects in maintaining ecosystem functioning for the QTP grasslands. But surprisingly, the optimum species diversity-productivity relationship shows a two-stage pattern, in which, with increasing species diversity, communities’ productivity increases non-significantly at low diversity levels but significantly at middle and high levels. The pattern is opposite to general relationships (e.g., positively linear, logarithmic, unimodal, or neutral), and thus it cannot be explained by common hypotheses such as rivet, compensatory/keystone species, redundant species, or null hypothesis (O’Connor and Crowe, 2005; Kang et al., 2015), but rather a unique mechanism potentially adapted to plant communities along environmental pressure gradients. The mechanism emphasizes a species diversity threshold, and below it, extreme environmental conditions (e.g., extreme low temperatures and intense radiation in some alpine zones, and low soil nutrients and drought stress in desert steppe zones) filter out most species, resulting in the coexistence of few species. The life history strategy of surviving species is to adapt to these environments, resulting in the lack of strong interaction and significant niche complementarity among them. Thus, the biomass production of these communities may be largely determined by restrictive resources or environmental factors, rather than by species composition and diversity. In other communities, no resources are significantly limited, which allows the coexistence of multiple species with different ways of resource utilization. Increasing species diversity will enhance plant use efficiency of different resources, and ultimately increase community productivity (Liu et al., 2015; Duffy et al., 2017). These results, meantime, suggest the common finding that facilitative plant interactions drive high-altitude plant diversity and every species contributes to community functioning, especially when species diversity is high (Maestre et al., 2009; Michalet et al., 2014). On the contrary, the best model for explaining the functional diversity-productivity relationship for most traits is positively linear, confirming the general prediction that functional differences among coexisting species are the basis of niche complementarity, and a great inter-specific functional difference will enhance community-level resource utilization efficiency (Wagg et al., 2017; Mensah et al., 2018).

Contrary to our predictions (hypothesis 2) and most previous studies (Cadotte et al., 2009; Chalcraft et al., 2009; Lemoine et al., 2015; Liu et al., 2015; Rolo et al., 2016; Zhu et al., 2016; Rosenfield and Müller, 2020), phylogenetic and functional diversity explain less biomass variation of the QTP grassland communities than species richness. Strong environmental filtering effect on functional traits may have caused this inconsistency (Kraft et al., 2007; Rolo et al., 2016). In general, only fewer evolutionarily related species with similar morphological features can survive under stressful environments. In this case, species’ functional traits may better predict their response to environmental stress but not their community status and relationships to other species (Schöb et al., 2013; He and Bertness, 2014). For most QTP grassland communities, plants live under rich soil nutrition and light resources but harsh climate conditions. As a result, species’ adaption to climate, rather than interspecific competition for resources, may be the primary force structuring these communities, resulting in the decoupling of species traits and community function to a certain extent (Schmitz et al., 2015; Cadotte, 2017). Meantime, our result implies the importance of species diversity in maintaining the function of some special ecosystems even though coexisting species show similar morphological traits.

### The Relationship Among Different Diversity Metrics

Consistent with hypothesis 3, our best model for explaining the species’ and functional or phylogenetic diversity relationship for the QTP grasslands is non-linear, in which functional or phylogenetic diversity (SES_*FD*_ of muli-traits and SES_*M*_) increases significantly with species richness after it exceeds a threshold. The result demonstrates that high functional and phylogenetic divergence, representing significant species’ niche differentiation or interspecific difference (dissimilarity) in resource acquisition or other life-history strategies (Violle et al., 2007; Bu et al., 2014; Rolo et al., 2016; Lyu et al., 2017), is important to maintain species coexistence in high species diversity communities. In low species diversity communities, however, interspecific competition for resource and (or) interspecific life history differences are not strong determinants of species coexistence, and as a result, species’ niche differentiation may be determined mainly by stochastic processes, leading to less dependence of average trait divergence (dissimilarity) to species diversity.

It has been frequently reported that two opposite niche processes, species interaction and habitat filtering, may not be mutually exclusive but instead operate simultaneously across grassland communities (Anthelme et al., 2014; Michalet et al., 2014; Zhu et al., 2016). Quantile regression for the functional species diversity relationship may help to disentangle their effects in structuring community, in which the upper (or lower) bound should represent the maximum competitive effect (or filtering effect) due to the higher (or lower) functional divergence among species. Our result, however, shows that the relationship changed across quantiles, shifting from a significantly and steeply positive slope in the lower quantile to a slightly positive or negative slope for a single trait and even a near-zero for multi-traits or phylogeny in the upper quantile. This suggests a filled triangle for the limit of the bivariate distribution, implying that functional diversity differs significantly among communities when their species diversity is low but finally converges to be a constant with increasing communities’ species diversity. The reason for big functional diversity differences for low-species diversity communities may be that they are dominated by strong species competition, which excludes low-competitive species and increases inter-specific trait dissimilarity, or alternatively, by strong habitat filtering, which excludes low-adaptive species and increases inter-specific trait similarity. By decreasing the effect of species competition or habitat filtering, more species can coexist, and these opposite niche processes may operate simultaneously and tend to balance each other (He and Bertness, 2014; Fichtner et al., 2017). Accordingly, a constant interspecific trait divergence (similar to limiting similarity theory; Kembel and Hubbell, 2006), representing the result of species’ response to the combined effect of opposite niche processes, enables the community to adapt to the fluctuations of environment, to sufficiently use local resources, and to keep community productivity at a high level.

### The Diversity-Functionality Relationship of Different Traits

According to the best model, we show significantly different patterns in the relationship between functional diversity of plant height, leaf and seed traits and community productivity and (or) species diversity, suggesting the different roles of these traits in structuring the QTP grasslands. Leaf traits (leaf size and SLA) significantly contribute to achieving a predictive framework for community species diversity and ecosystem functioning. Leaf traits are tightly related to light resource acquisition and plant growth strategy and identified as a key functional predictor of the plant capacity of adaptation to changeful and stressful environments. The result is in accordance with previous studies suggesting that the diversity pattern of leaf traits, especially SLA, can be used to predict communities’ productivity, carbon storage and assembly rule (Long et al., 2011; Lemoine et al., 2015). By contrast, the relationships between diversity patterns of plant height and seed mass and community species diversity and productivity are generally weak or non-significant, implying that plant’s light interception capability and seedling establishment’s ability may be less important in structuring QTP grassland communities with a rich light resource for under plants. Moreover, most Tibetan grassland species are typically rosette (or semi-rosette) plants or primarily reproduce asexually (Nagy and Grabherr, 2009; Qi et al., 2014; Chen et al., 2017). These unique plants’ architectural and reproductive traits would weaken the effects of plant height and seed mass on supporting leaf light interception and enhancing seedling establishment and survival, respectively, and thus diminish their roles in driving community assembly. In addition, the piecewise model shows that communities with lowest functional diversity of leaf traits usually have no lowest productivity (Table 1, Figure 3), implying that, in some special zones (e.g., desert steppe) where light competition is not severe, similar leaf traits may be more helpful for coexisting species to adapt to the environment and use various resources.

## Conclusion

Across the QTP grassland communities, we showed an overall positive biodiversity-productivity relationship in species’, functional and phylogenetic space. The relationship, however, was non-linear, in which increasing species diversity, communities’ productivity increased significantly at middle and high diversity levels but not at the low level, suggesting a weak effect of biodiversity on ecosystem function in low species diversity communities. In the meantime, phylogenetic and functional diversity explained less biomass variation of the grassland communities than species richness, highlighting species diversity in maintaining grassland community stability and ecosystem functions. We also found a filled triangle for the limit of the relationship between species and functional diversity, implying that functional diversity differs significantly among communities when their species diversity is low but finally converges to be a constant with increasing communities’ species diversity. The above findings suggest that multiple niche processes may structure the grassland communities, and their forces tend to balance in high-biodiversity communities.

## Data Availability Statement

The original contributions presented in the study are included in the article/Supplementary Material, further inquiries can be directed to the corresponding author/s.

## Author Contributions

XK, WQ, GD, and JK conceived the ideas. All authors but XK and JK collected the data. XK and WQ analyzed the data. XK, WQ, and JK contributed to the writing of the manuscript. All authors contributed to the article and approved the submitted version.

## Conflict of Interest

The authors declare that the research was conducted in the absence of any commercial or financial relationships that could be construed as a potential conflict of interest.

## Publisher’s Note

All claims expressed in this article are solely those of the authors and do not necessarily represent those of their affiliated organizations, or those of the publisher, the editors and the reviewers. Any product that may be evaluated in this article, or claim that may be made by its manufacturer, is not guaranteed or endorsed by the publisher.

## Footnotes

1
https://github.com/camwebb/phylomatic-awk

## Supplementary Material

Supplementary Figure 1 Mean (± 95% CI) standardized effect size (SES) of functional diversity (FD_*Q*_) for single- and multi-traits and of phylogenetic diversity in the Tibetan grassland communities. Symbols in black indicate values significantly different from zero.

Supplementary Figure 2 SES of FD_*Q*_ for leaf size **(A)**, specific leaf area (SLA, **B**), plant height **(C)**, seed mass **(D)**, multiple traits **(E)**, and phylogenetic diversity (MPD, **F**) in relation to the Shannon–Wiener index (H, x-axis) at the upper (95th), median (50th), and lower (5th) quantile levels. Significant and non-significant linear relationships (at α = 0.05) were shown as solid and dashed lines, respectively.

Supplementary Appendix 1 A comparison of location, climate attributes, and community characters of the 118 study sites. “Richness” and “Abundance” represent species number and total individual number in a 1 m^2^ quarter.
