# Supplementary figures and images for: The Complex Biodiversity-Ecosystem Function Relationships for the Qinghai-Tibetan Grassland Community
Source: Front Plant Sci. 2022 Jan 27;12:772503. doi: 10.3389/fpls.2021.772503 (PMC8829388; doi:10.3389/fpls.2021.772503)

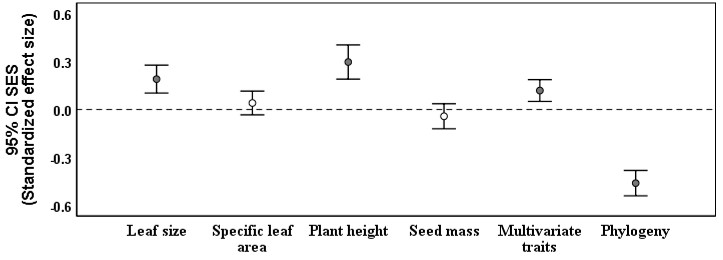

Supplement: Supplementary Figure 1 — Mean (± 95% CI) standardized effect size (SES) of functional diversity (FDQ) for single- and multi-traits and of phylogenetic diversity in the Tibetan grassland communities. Symbols in black indicate values significantly different from zero. [file Image_1.jpg]

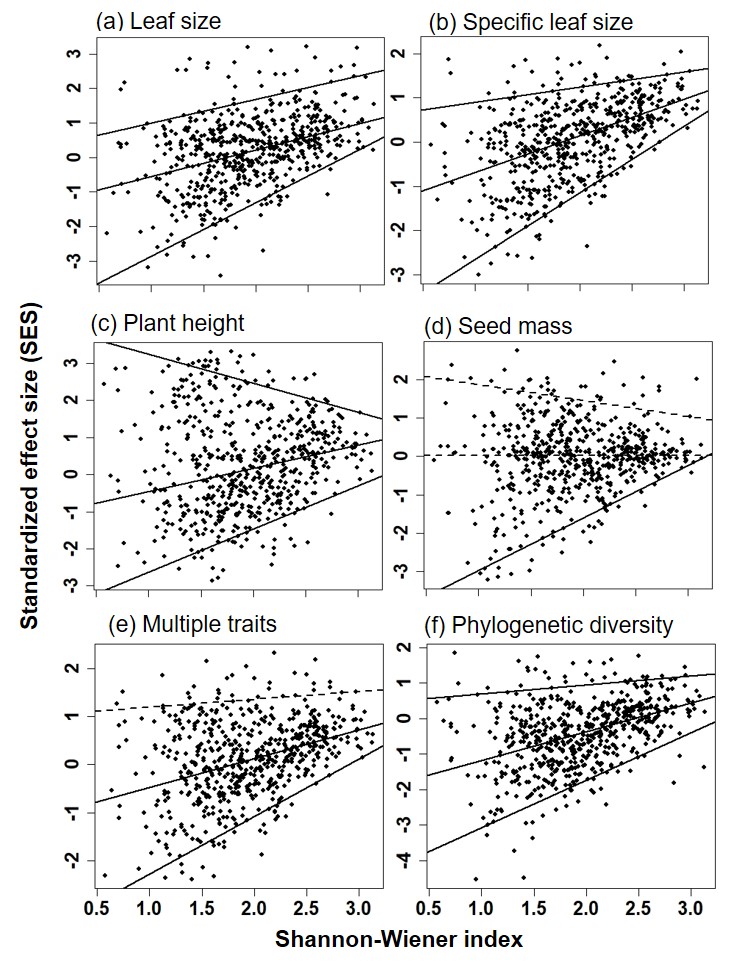

Supplement: Supplementary Figure 2 — SES of FDQ for leaf size (A), specific leaf area (SLA, B), plant height (C), seed mass (D), multiple traits (E), and phylogenetic diversity (MPD, F) in relation to the Shannon–Wiener index (H, x-axis) at the upper (95th), median (50th), and lower (5th) quantile levels. Significant and non-significant linear relationships (at α = 0.05) were shown as solid and dashed lines, respectively. [file Image_2.jpg]
