# Supplementary material for: The Complex Biodiversity-Ecosystem Function Relationships for the Qinghai-Tibetan Grassland Community
Source: Front Plant Sci. 2022 Jan 27;12:772503. doi: 10.3389/fpls.2021.772503 (PMC8829388; doi:10.3389/fpls.2021.772503)
Supplement: Supplementary file 4 [file Table_1.doc]

**(A) Species richness (x-axis) vs. functional or phylogenetic diversity indexes**

| Diversity indexes (y-axis) | Lower quantile (5th) | | |  | Median quantile (50th) | | |  | Upper quantile (95th) | | |
| --- | --- | --- | --- | --- | --- | --- | --- | --- | --- | --- | --- |
|  | a | b | Sig. |  | a | b | Sig. |  | a | b | Sig. |
| SES_FD_ of leaf size | **3.922** | **-5.582** | **<0.001** |  | **2.008** | **-2.027** | **<0.001** |  | **1.601** | **-0.058** | **<0.001** |
| SES_FD_ of SLA | **4.017** | **-5.493** | **<0.001** |  | **2.186** | **-2.324** | **<0.001** |  | **1.110** | **-0.006** | **0.002** |
| SES_FD_ of plant height | **1.958** | **-3.648** | **<0.001** |  | **1.404** | **-1.390** | **<0.001** |  | **-1.718** | **4.362** | **<0.001** |
| SES_FD_ of seed mass | **3.684** | **-5.654** | **<0.001** |  | -0.098 | 0.138 | 0.450 |  | **-1.231** | **2.741** | **<0.001** |
| SES_FD_ of multi-traits | **3.343** | **-4.708** | **<0.001** |  | **1.330** | **-1.331** | **<0.001** |  | 0.344 | 0.950 | 0.171 |
| SES_M_ (SES of MPD) | **3.367** | **-5.430** | **<0.001** |  | **2.801** | **-2.774** | **<0.001** |  | **0.663** | **0.213** | **0.032** |

**(B) Shannon–Wiener index (H, x-axis) vs. functional or phylogenetic diversity indexes**

| Diversity indexes (y-axis) | Lower quantile (5th) | | |  | Median quantile (50th) | | |  | Upper quantile (95th) | | |
| --- | --- | --- | --- | --- | --- | --- | --- | --- | --- | --- | --- |
|  | a | b | Sig. |  | a | b | Sig. |  | a | b | Sig. |
| SES_FD_ of leaf size | **1.556** | **-4.420** | **<0.001** |  | **0.769** | **-1.324** | **<0.001** |  | **0.690** | **0.311** | **0.017** |
| SES_FD_ of SLA | **1.506** | **-4.162** | **<0.001** |  | **0.830** | **-1.516** | **<0.001** |  | **0.344** | **0.558** | **0.008** |
| SES_FD_ of plant height | **1.159** | **-3.788** | **<0.001** |  | **0.623** | **-1.074** | **<0.001** |  | **-0.781** | **4.029** | **0.001** |
| SES_FD_ of seed mass | **1.372** | **-4.352** | **<0.001** |  | 0.001 | 0.030 | 1.000 |  | -0.416 | 2.296 | 0.074 |
| SES_FD_ of multi-traits | **1.199** | **-3.478** | **<0.001** |  | **0.596** | **-1.064** | **<0.001** |  | 0.165 | 1.033 | 0.149 |
| SES_M_ (SES of MPD) | **1.339** | **-4.411** | **<0.001** |  | **0.813** | **-1.998** | **<0.001** |  | **0.256** | **0.430** | **0.039** |

**Table S1.** The binary relationship between communities’ species richness (A, x-axis, log-scale) or Shannon–Wiener index (B, x-axis) and their functional or phylogenetic diversity indexes (y-axis) at lower (5th), median (50th) and upper (95th) quantile for the Tibetan grasslands. The regression equation for the binary relationship was calculated as: y = a*x + b, in which a and b represented the slope and intercept of the equation, respectively. The significantly binary relationship was shown in bold.
