# Supplementary material for: The Complex Biodiversity-Ecosystem Function Relationships for the Qinghai-Tibetan Grassland Community
Source: Front Plant Sci. 2022 Jan 27;12:772503. doi: 10.3389/fpls.2021.772503 (PMC8829388; doi:10.3389/fpls.2021.772503)
Supplement: Supplementary file 6 [file Data_Sheet_2.doc]

The methods of functional trait measurement

In our study, specific leaf area (SLA) and leaf area were measured for each species in each site on at least five (two for very large leaves and at least ten for very small leaves) randomly chosen, fully developed but not senescing leaves from different individuals. All leaves were placed on the scanner to avoid overlap and fully expand bent or contracted leaves. Leaf area was calculated by image analysis software (Scion Image 4.0.2). All imaged leaves were dried at 65 °C to a constant mass and weighed to the nearest 0.001 g. Compound leaf was measured here as an entire leaf. However, about 46% data were not measured in each site, respectively, because the software cannot accurately evaluate the area of linear leaves with leaf width < 2 mm or for other reasons. Part of missing SLA and leaf area values were obtained from the sites with similar altitude and habitat of this studies, or alternatively, from the studies in the same area (He et al. 2006, Yang et al. 2012, Qin et al. 2014, Qi et al. 2014a, 2015a, Elumeeva et al. 2015). For remaining missing SLA value, it was replaced by the average value of the species group with the same leaf texture (membranous, chartaceous, rigidly chartaceous or subcoriaceous, coriaceous), leaf thickness (very thin, about <0.1 mm; thin, 0.1-0.2 mm; moderate, about 0.2-0.3 mm; thick, about > 0.3 mm) and leaf pubescence (none/scattered, dense) from a large database (altogether 18 species groups, including 1071 species, data not shown). The 18 groups accounted for 70.1% of the interspecific SLA variation, and mean interspecific variation within groups was near 8-times lower than interspecific variation across all species. Thus, the method is suitable for estimating missing SLA data. For remaining missing leaf area value, it was evaluated from the *Flora of China* (Wu & Raven 1994-2009, also see *Flora Reipublicae Popularis Sinicae*, <www.efloras.org>), *Flora of Qinghai* (Liu 1996-1999), *Flora of Tibet* (Wu 1983-1987) and *Higher Plants of China* (Fu et al. 1999-2005) according to leaf shape, average leaf length and leaf width.

For most (83.3%) species, plant height was evaluated in the field. About 3-5 mature individuals were randomly sampled for each species and measured along the main stem from the base to the highest point on the plant (including reproductive parts). Plant height of remaining species was compiled from *Flora of China* (Wu & Raven 1994-2009), *Flora of Qinghai* (Liu 1996-1999), *Flora of Tibet* (Wu 1983-1987) and *Higher Plants of China* (Fu et al. 1999-2005). Moreover, the height of small rosette plants (93 species) was recorded as 1 cm to meet the requirement of calculating community functional diversity.

Seed mass was defined as the weight of the embryo and endosperm, plus the seed coat. Other structures contributing to dispersal were not included as part of the seed. Seed mass of 66% species in the study were from a large database (approx. 1570 species and 11000 populations) based on our previous studies (see Liu et al. 2011, 2013, Qi et al. 2014b, 2015b, Bu et al. 2016, Xu et al. 2017, Zhang et al. 2017, and unpublished data). In the database, seeds were gathered in late summer and autumn during 2002-2011 and were collected at the start of natural dispersal. In every winter, seeds were air-dried to a constant mass at room temperature (approximately 15 ℃), and then weighed 100 seeds from pooled collections three times for each population of each species whenever possible. The missing seed mass value was obtained from the studies in the same area (Wang et al. 2012, Niu et al. 2014), or alternatively, was calculated according to known seed mass of congeneric or closely related species with similar seed shape. Two congeneric species, for example, both had spherical seeds, in which the average diameter and mass of one species’ seeds was 5 mm and 1 mg, respectively. The average seed mass of the other species with 6 mm seed diameter could be estimated as 1.728 mg (6^3^/5^3^ * 1 mg). The method was well suitable for estimating missing seed mass data because the average difference between estimated and actual seed mass value of 242 known generic species pairs was lower than 9%.

References

Bu H, Wang XJ, Zhou XH, Qi W, Liu K, Ge W, Xu D, Zhang S (2016) The ecological and evolutionary significance of seed shape and volume for the germination of 383 species on the eastern Qinghai-Tibet plateau. Folia geobotanica, 51(4): 333-341.

Elumeevaa TG, Onipchenkoa VG, Yan W (2015) Leaf Functional Traits of Plants of Alpine Pastures at the Eastern Qinghai-Tibetan Plateau. Moscow University Biological Sciences Bulletin, 70(1): 46-52.

Fu G et al., eds. (1999-2005) Higher plants of China. Qingdao Publishing House.

He J, Wang Z,. Wang X, Schmid B, Zuo W, Zhou M, Zheng C, Wang M, Fang J (2006) A test of the generality of leaf trait relationships on the Tibetan Plateau. New Phytologist, 170: 835-848.

Liu K, Baskin JM, Baskin CC, Bu H, Liu M, Liu W, Du G (2011) Effect of storage conditions on germination of seeds of 489 species from high elevation grasslands of the eastern Tibet Plateau and some implications for climate change. American Journal of Botany, 98(1): 12-19.

Liu K, Baskin JM, Baskin CC, Bu H, Du G, Ma M (2013) Effect of diurnal fluctuating versus constant temperatures on germination of 445 species from the eastern Tibet Plateau. Plos One, 8: e69364.

Liu S (1996-1999) Flora of Qinghai (vol. 1-4). Qinghai People Press, Xining.

Niu K, Choler P, de Bello F, Mirotchnick N, Du G, Sun S. (2014) Fertilization decreases species diversity but increases functional diversity: a three-year experiment in a Tibetan alpine meadow. Agriculture, ecosystems & environment, 182: 106-112.

Qi W, Bu H, Liu K, Li W, Knops JMH, Wang J, Li W, Du GZ (2014a) Biological traits are correlated with elevational distribution range of eastern Tibetan herbaceous species, Plant Ecology, 215: 1187-1198.

Qi W, Guo S, Chen X, Cornelissen JHC, Bu H, Du G, Cui X, Li W, Liu K (2014b) Disentangling ecological, allometric and evolutionary determinants of the relationship between seed mass and elevation: insights from multiple analyses of 1355 angiosperm species on the eastern Tibetan Plateau, Oikos, 123(1): 23-32.

Qi W, Zhou X, Ma M, Knops JMH, Li W, Du G (2015a) Elevation, moisture and shade drive the functional and phylogenetic meadow communities’ assembly in the northeastern Tibetan Plateau, Community Ecology, 16(1): 66-75.

Qi W, Bu H, Cornelissen JHC, Zhang C, Guo S, Zhou X, Li W, Du G (2015b) Untangling interacting mechanisms of seed mass variation with elevation: insights from the comparison of inter-specific and intra-specific studies on eastern Tibetan angiosperm species, Plant Ecology, 216: 283-292.

Qin Y, Yi S, Ren S, Li N, Chen J (2014) Responses of typical grasslands in a semi-arid basin on the Qinghai-Tibetan Plateau to climate change and disturbances. Environmental Earth Sciences, 71: 1421-1431.

Wang J, Chen W, Baskin, CC, Baskin JM, Cui X, Zhang Y, Qiang W, Du G (2012) Variation in seed germination of 86 subalpine forest species from the eastern Tibetan Plateau: phylogeny and life-history correlates. Ecological research, 27: 453-465.

Wu Z (1983-1987) Flora of Xizang. Science Press, Beijing.

Wu Z, Raven PH, eds. (1994-2009) Flora of China. Science Press & Missouri Botanical Garden Press, Beijing & St Louis.

Xu J, Li W, Zhang C, Liu W, Du G. (2017) The determinants of seed germination in an alpine/subalpine community on the Eastern Qinghai-Tibetan Plateau. Ecological Engineering, 98: 114-122.

Yang Z, Jeff RP, Zhang C, Du G (2012) The effect of environmental and phylogenetic drivers on community assembly in an alpine meadow community. Ecology, 93: 2321-2328.

Zhang C, Ma Z, Du G (2017) Light-dependent associations of germination proportion with seed mass in alpine grasslands of the Qinghai-Tibet plateau. Ecological Engineering, 105: 306-313.
