# Supplementary material for: The Complex Biodiversity-Ecosystem Function Relationships for the Qinghai-Tibetan Grassland Community
Source: Front Plant Sci. 2022 Jan 27;12:772503. doi: 10.3389/fpls.2021.772503 (PMC8829388; doi:10.3389/fpls.2021.772503)
Supplement: Supplementary file 7 [file Data_Sheet_3.doc]

The phylogenetic signals of plant functional traits and their calculation method

Method

We examined the phylogenetic signal strength by estimating Pagel’s λ for each trait using "ﬁtContinuous" functions in the R package "geiger" (Harmon *et al.* 2008; Münkemüller *et al.* 2015). We used a maximum likelihood framework to estimate the parameter λ (phylogenetic signal value), which can vary from 0 (no inﬂuence of phylogeny) to 1 (maximum phylogenetic inﬂuence). Approximate confidence intervals for the maximum likelihood value of λ were calculated via likelihood ratio tests (Freckleton *et al.* 2002). We still used ‘R20120829’ version available at http://www.phylodiversity.net as basal tree to generate the working phylogenetic tree of this analysis.

Results

Phylogenetic signal across a phylogeny of 719 east and center Tibetan grassland species were shown as following table. In the table, the maximum likelihood value of λ was given along with confidence intervals for this value, and the significance (*P*-values) of the difference from λ = 0 and λ = 1.

| Functional traits | λ | 95% CI | Significance of the difference from | |
| --- | --- | --- | --- | --- |
|  |  |  | λ = 0 | λ = 1 |
| Leaf size | 0.41 | 0.24-0.66 | < 0.001 | < 0.001 |
| Specific leaf area | 0.67 | 0.46-0.79 | < 0.001 | < 0.001 |
| Plant height | 0.32 | 0.13-0.58 | 0.001 | < 0.001 |
| Seed mass | 0.98 | 0.96-0.99 | < 0.001 | < 0.001 |

According to above table, we found a significant phylogenetic signal in all functional traits (λ significantly different from 0), with the stronger signal for seed mass, followed by specific leaf area, leaf size and plant height. Based on the λ value, there were high phylogenetic conservatism in seed mass (λ = 0.98) and specific leaf area (λ = 0.67), but low in leaf size (λ = 0.41) and plant height (λ = 0.32).

References

Freckleton, R.P., Harvey, P.H. & Pagel, M. (2002). Phylogenetic analysis and comparative data: a test and review of evidence. *Am. Nat.*, 160, 712-726.

Harmon, L.J., Weir, J.T., Brock, C.D., Glor, R.E. & Challenger W. (2008). GEIGER: investigating evolutionary radiations. *Bioinformatics*, 24, 129-131.

Münkemüller, T., Boucher, F.C., Thuiller, W. & Lavergne, S. (2015). Phylogenetic niche conservatism—Common pitfalls and ways forward. *Funct. Ecol.*, 29, 627-639.
