# Supplementary material for: The Complex Biodiversity-Ecosystem Function Relationships for the Qinghai-Tibetan Grassland Community
Source: Front Plant Sci. 2022 Jan 27;12:772503. doi: 10.3389/fpls.2021.772503 (PMC8829388; doi:10.3389/fpls.2021.772503)
Supplement: Supplementary file 8 [file Data_Sheet_4.docx]

### Results of the difference in species’, functional and phylogenetic diversity among six grassland types

1. Statistical analyses

We used one-way ANOVA to test for the difference among six grassland types in their aboveground biomass (AGB, log-scale), species richness (SR, log-scale) and Shannon–Wiener index (H), phylogenetic diversity index (SES_M_, standardized effect size of mean pairwise phylogenetic distance), and various functional diversity indexes [SES_FD_ of leaf size, specific leaf area (SLA), plant height, seed mass and multiple traits; SES_FD_ represents the standardized effect size for Rao's quadratic index (FD_Q_) of functional traits].

1. Results

Based on Figure 1, meadow (desert steppe) had highest (lowest) SR, H and AGB. Steppe and wet meadow had lower SR and H, but medium (for steppe) or higher (for wet meadow) AGB. Moreover, steppe meadow and cushion vegetation had medium SR, H and AGB.


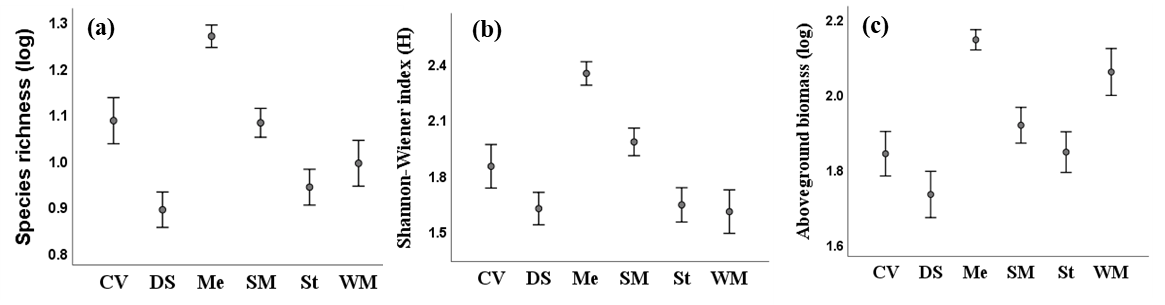


**Fig. 1.** The difference among six grassland types in their species richness (a, species/m^2^), Shannon–Wiener index (b) and aboveground biomass (c, g/m^2^). CV, cushion vegetation; DS, desert steppe; Me, meadow; SM, steppe meadow; St, steppe; WM, wet meadow.

Based on Figure 2, meadow had highest SES_M_ (non-significant > 0) and SES_FD_ of leaf size, SLA and multiple traits (all significant > 0), but medium SES_FD_ of plant height (significant > 0) and seed mass (non-significant < 0). Steppe meadow had higher SES_FD_ of leaf size, SLA seed mass and multiple traits (all significant > 0), but medium SES_M_ (significant < 0) and SES_FD_ of plant height (significant > 0). Steppe had highest SES_FD_ of plant height and seed mass (both significant > 0), higher SES_FD_ of multiple traits (significant > 0), and medium SES_M_ (significant < 0) and leaf traits (no different from 0). Cushion vegetation had lowest SES_FD_ of leaf size, plant height and multiple traits (all significant < 0), lower SES_FD_ of SLA (significant < 0), medium SES_FD_ of plant height (significant > 0) and higher SES_M_ (non-significant < 0). Desert steppe had lowest SES_FD_ of SLA (significant < 0), lower SES_M_ and SES_FD_ of leaf size (both significant < 0), medium SES_FD_ of plant height (significant > 0) and multiple traits (non-significant < 0), but higher SES_FD_ of seed mass (significant > 0). Instead, wet meadow had lowest SES_M_ and SES_FD_ of seed mass (both significant < 0), lower SES_FD_ of leaf traits and multiple traits (all significant < 0), but medium SES_FD_ of plant height (non-significant < 0).

**
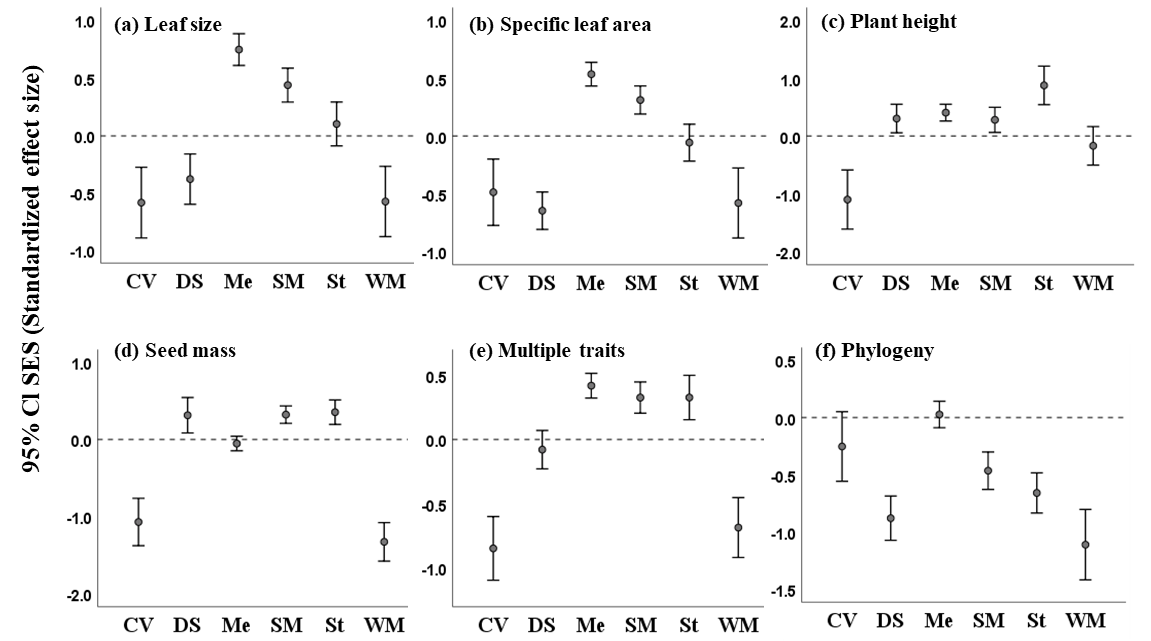
**

**Fig. 2.** The difference among six grassland types in functional diversity of different traits (a-e; i.e., SES_FD_ of leaf size, specific leaf area (SLA), plant height, seed mass and multiple traits, respectively) and phylogenetic diversity (f; SES_M_). CV, cushion vegetation; DS, desert steppe; Me, meadow; SM, steppe meadow; St, steppe; WM, wet meadow.
